# “The Competitive Season and Off-Season”: Preliminary Research concerning the Sport-Specific Performance, Stress, and Sleep in Elite Male Adolescent Basketball Athletes

**DOI:** 10.3390/ijerph182413259

**Published:** 2021-12-16

**Authors:** Chun-Chung Chou, Fei-Ti Wang, Hsin-Hung Wu, Shiow-Chwen Tsai, Chung-Yu Chen, Jeffrey R. Bernard, Yu-Chi Kuo, Yi-Hung Liao

**Affiliations:** 1 Physical Education Office, National Taipei University of Technology, Taipei 10608, Taiwan; longer0206@gmail.com; 2 Department of Exercise and Health Science, National Taipei University of Nursing and Health Sciences, Taipei 11219, Taiwan; micky1726@gmail.com (F.-T.W.); jazzman0410@gmail.com (H.-H.W.); 3 Department of Neurology, Cheng Hsin General Hospital, Taipei 11219, Taiwan; 4 Institute of Sports Sciences, University of Taipei, Taipei 11153, Taiwan; sctsai6@gmail.com; 5 Department of Exercise and Health Sciences, University of Taipei, Taipei 11153, Taiwan; fish0510@gmail.com; 6 Department of Kinesiology and Public Health Promotion, California State University, Stanislaus, Turlock, CA 95382, USA; jbernard1@csustan.edu

**Keywords:** dehydroepiandrosterone sulfate (DHEA-S), cortisol, sleep quality, mood state, change of direction

## Abstract

Background: Through scholastic sports programs, adolescent athletes compete to represent their communities. However, few studies investigate the changes in physiological and mental profiles during varied sport periodization among this population. Therefore, the purpose of this study was to compare the changes in sports performance and stress-related biomarkers between the competitive season (CS) and off-season (OS) in elite adolescent basketball players. Method: Nine elite Division I male basketball players (age: 15–18 years. old) participated in this study. Basketball-specific performance, salivary dehydroepiandrosterone sulfate (DHEA-S)/cortisol levels, mood state, and sleep quality were all accessed during the CS and OS periods. Results: The training load during OS was 26.0% lower than CS (p = 0.001). Muscle mass, aerobic capacity, 10 m sprint, and Abalakov jump (AJ) power during OS were greater than that during CS (+2.2–9.8%, p < 0.05), but planned agility was greater during CS (p = 0.003). The salivary DHEA-S/cortisol was greater during CS than during OS (p = 0.039). The overall mood state and sleep quality did not differ between periods, but the POMS-tension was higher during CS (p = 0.005). Conclusion: The present study demonstrates that muscle mass, aerobic capacity, peak AJ power, and 10 m sprint performance, but not planned agility, were greater during OS compared to CS among elite adolescent basketball players. Furthermore, the stress-related responses reflected by the D/C ratio and mood tension were relatively lower during the OS in these athletes. Thus, this study suggests that coaches and sport science professionals should closely monitor athletes’ training states across varied training/competition periods to better react to modifying training or recovery plans.

## 1. Introduction

High school student–athletes spend a great deal of time meeting the physical and competition preparation demands of their sport training, which includes physical training, strength training, simulated game practice, and official competition throughout the season. At the same time, high school student–athletes also require ongoing physical and skill training during the off-season, and student–athletes also need to cope with their academic workloads. It is well known that participation in intensive sports training and league play affects a variety of physiological parameters in collegiate athletes [1,2], including body composition, muscle strength, and sports performance characteristics. However, there is a lack of studies that have examined the potential changes in these parameters as youth elite basketball players move from the competitive to the off-season.

The student–athletes stand out among their peers. The typical student has their basic academic requirements. However, student–athletes not only have the basic academic requirements but the additional training and competition associated with scholastic sports, making this is a particularly challenging and oftentimes stressful balancing act between academics and sports for adolescent athletes. Unlike more studied populations, such as collegiate or professional athletes [3,4,5], adolescent student–athletes may be undergoing various stages of puberty. Thus, the internal physiological/mental changes associated with puberty are compounded by the external stresses of schoolwork and athletic training and competition [6,7]. Therefore, students need to develop routines to attenuate external stresses while the internal stresses of physiological/mental development continue. Unfortunately, studies investigating the stress status placed on student–athletes are limited. Although a current systemic review reveals that regular participation in high-intensity interval exercise would positively affect cognitive performance and psychological factors [8], there is even less known about the changes in physiological and mental profiles during varied sports periodization in adolescent athletes.

Training periodization refers to athletic training that is structured around periods of progressive overload training followed by periods of rest [9]. According to sport-specific training and competitive performance, athletes’ physiological and psychological parameters may vary at the different periods during their annual training plan [1,2,10,11,12,13]. During the off-season (the end of one season until the next), high school basketball players typically participate in a regular physical training program designed to optimize agility, speed, aerobic, and anaerobic capacity. During the competitive season, strength and conditioning are substituted for team training, simulated games, friendly competition, and athletic recovery. On the other hand, unlike professional athletes, student–athletes may not devote all their time to training periodization. A way to circumvent this, while still reaching peak athletic performance, is to increase training volume and practice time [14]. However, this puts tremendous pressure and stress on student–athletes, both athletically and academically, which may increase the likelihood of illness or injury [11,12,13] and negatively affect their sleep, recovery, physiological adaptations [15], and academic performance [16]. For example, impaired sleep quality and quantity appear to cause athletes to develop symptoms similar to those of overtraining syndrome; in addition, sleep deprivation affects cognitive function and may lead to slower and less accurate cognitive performance [17]. It has been reported that feelings of tiredness among adolescents oftentimes manifest themselves as difficulty maintaining behaviors that require effort [18] and increase the risk of sport injuries during training/competition [13,19]. Moreover, several recent systemic reviews have revealed that changes in salivary markers (e.g., cortisol, testosterone, immunoglobulin A, total protein) were observed during long and short training periods in basketball players and that basketball games also induce highly stressful salivary marker changes [20,21]. However, few studies have explored the relationship between changes in stress hormone concentrations, specific physical performance, and sleep quality among varied training/competition periods in adolescent elite players, particularly in basketball. It is important to closely monitor the changing patterns of these stress-related biomarkers and sleep quality during both competitions and the off-season periods to better prescribe the appropriate training/recovery programs for adolescent basketball athletes.

We hypothesized that overall, sports performance, conditioning, and body composition would be better during the competitive season than during the off-season period. As a result of the substantial diversity in training volume and target demands between the competitive and off-season periods, we further hypothesized that sleep quality, fatigue status, and anabolic metabolic stress biomarkers might be affected by the transition from competitive season to the off-season period in elite high school basketball players. Therefore, this study investigated differences in the salivary stress-related hormonal response, mood states, sleep quality, and basketball-specific physical performance between the competitive season (CS) and off-season (OS) periods in elite adolescent basketball players. These findings may help coaches and sports scientists to better understand the changes in mental and physiological stress states among elite adolescent basketball players.

## 2. Materials and Methods

### 2.1. Study Design

The purpose of this study was to investigate the periodic changes in sport performance, biomarkers, mood state, and sleep quality in elite high-school basketball athletes. Therefore, the present study design was observational in nature, with no control group. Elite male adolescent Division I high school basketball players were recruited in this study, and our study design is to observe the changes of salivary stress-related hormonal response, mood states, sleep quality, and basketball-specific physical performance between the competitive season (CS) and off-season (OS) periods in elite adolescent basketball players. This study was approved by the Institutional Review Board (IRB) of National Yang Ming University (protocol#: YM105088F) and performed following the current Declaration of Helsinki.

### 2.2. Participants

Nine elite male adolescent Division I high school basketball players (aged 15–18 years) participated and completed all the tests/questionnaires in this study. Under their coaches’ supervision, participants maintained a year-round training schedule. All participants, as well as their guardians/parents, completed an informed consent form prior to the study. The athletes were from teams ranked among the top 8 in the nation at the Division I level. Thus, the athletes that participated in this study were categorized as elite. The anthropometric data of participants are shown in Table 1.

### 2.3. Procedure

**Figure 1:**
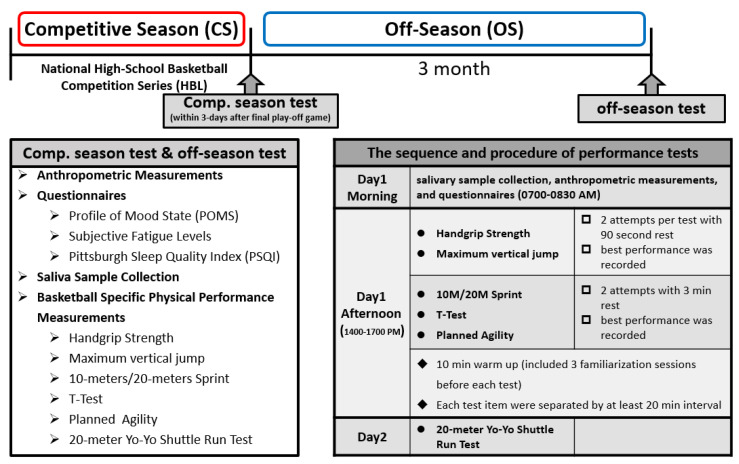
Diagram depicting measurement procedures for the competitive season and off-season.

Basic characteristics, physical performance, and sleep/mood state questionnaires were completed by the participating basketball players and collected during the national competitive season (National High-School Basketball Competition Series, High School Basketball League, Taiwan) and again at the end of the off-season recovery period. The training, consisting of basketball-specific skills and strength/conditioning practice during the periodic training program, was performed under their coaches’ supervision during both CS and OS periods (Table 2). All participants fully complied with the training programs. For the high school Division I basketball team in Taiwan, they were required to continue practicing until summer vacation; thus, the basketball players maintained their regular training during the OS period. For the experimental measurement procedure (study procedure/timeframe, see Figure 1), physical performance for the competition period was tested within 3 days after the last playoff game was completed (Pre-test), while the OS recovery period was tested 3 months after the national competition (Post-test). To minimize any possible impact from previous training sessions, strenuous exercise and resistance training were avoided for the 2 days leading into the physical performance test during the OS period (Post-test). Both Pre-test and Post-tests included anthropometric measurements, basketball-specific sports performance, a physical activity log, and mood/sleep quality questionnaires (Figure 1).

On the morning of the test day (0700–0830 AM), and following a 10 h fast, salivary samples and anthropometric measurements were collected. Participants also completed the questionnaires. During the afternoon on test day, all basketball-specific physical/sport performance tests were performed. The specific time of day was kept consistent throughout the study (1400–1700 PM). High-intensity exercise training, resistance training, or competitions were not permitted the two days prior to testing, thus ensuring consistency and minimizing possible confounding factors. The salivary samples were used primarily to analyze levels of the anabolic hormone (DHEA-S) and the stress hormone (cortisol) during the different training/competition periods.

### 2.4. Training and Diet Monitoring

From CS to OS periods, the participants were accommodated in student dorms, and the coaches standardized dietary plans and developed a monitored basketball-specific training program. The dietary plan was referenced to general recommendations (carbohydrate: ≈55%; protein: ≈25%; and fat: ≈20%). For training intensity, the basketball-specific skills and strength/conditioning practice were performed under their coaches’ supervision during both CS and OS periods, and the coach subjective rating training intensity (1–10; low-to-extremely high) is shown in Table 2. Moreover, the 3-day physical activity recall log (3d-PAL) was also used to record the level of physical activity during the CS and OS periods, as previously described [22].

### 2.5. Anthropometric Measurements

Two days before anthropometric measurements, all participants were asked to avoid any type of exercise, and the anthropometric measurements were performed after a 10 h overnight fast as previously described [22]. In brief, all participants were instructed to wear the same lightweight shorts and pants between different competition/off-season periods to ensure consistency in front-to-back testing. Bioelectrical impedance (BIA) (HBF-371, OMRON Inc., Kyoto, Japan) was used to determine body fat percentage and other anthropometric values (weight, body fat rate, fat-free weight, body mass index, etc.). When performing the BIA measurement, each measurement was taken after an overnight fast (10 h), and participants were asked to abstain from drinking water and any other liquids containing alcohol or caffeine for at least 4 h before the BIA assessment. Moreover, to ensure the accuracy of the body composition assessment, the BIA measurement was only taken between 08:00 and 09:00 a.m.

### 2.6. Basketball Specific Physical Performance Measurements

#### 2.6.1. Handgrip Strength

Dominant handgrip strength was measured using a digital hand-grip dynamometer (TKK 5401; Takei Scientific Instruments Co, Ltd., Tokyo, Japan). Participants took one attempt, rested for 90 s, and then made a second attempt. The best score was recorded and used for later analyses.

#### 2.6.2. Maximum Vertical Jump Performance

The Abalakov jump (AJ) test has been widely used to assess lower extremity jump power [23]. The test procedure followed that used in previous reports [24]. Briefly, participants began in an upright posture with feet shoulder-width apart. They were instructed to jump as high as possible, landing with both feet simultaneously. A 90 s rest was allowed between attempts. The best attempt was recorded and used for later analysis. The conversion for AJ height to peak vertical jump power value was calculated using the following formula [25]: peak power (Watts) = 61.9 × (jump height [cm]) + 36.0 × (body mass [kg]) + 1.822.

#### 2.6.3. 10 m/20 m Sprint Performance

Ten to 20 m sprint performance was measured using electronic timing gates (TCI Timing System, Brower Timing Systems, UT, USA) [24]. Timing gates were placed at the starting point (0 m; starting line), 10 m, and 20 m. The timing gate recorded 2 sprint session times at 10 m and 20 m, respectively. This set up allowed the researchers to measure speed and acceleration in 20 m. The recorded time (accuracy: 10 msec) was the best time obtained from two attempts, and the participants had a 3 min rest interval between 2 attempts.

#### 2.6.4. T-Test

**Figure 2:**
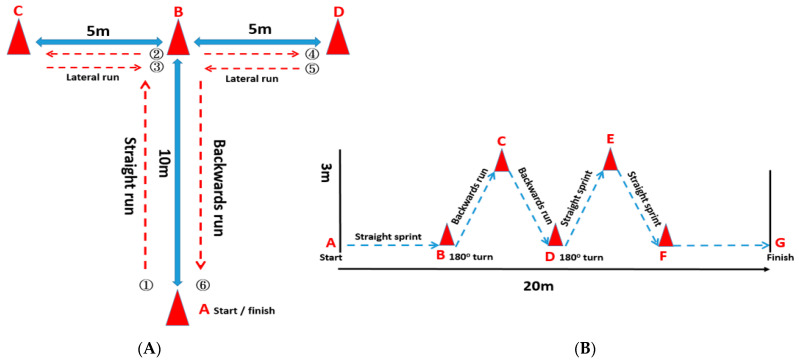
The procedure of the measurements at the competitive season and off-season. (**A**) The T-Test. (**B**) The planned agility test. The A → D and A → G represent the triangular cones used in the path of the T-Test and the planned agility test, respectively.

The T-Test was performed with slight modifications from those previously reported [26]. Briefly, four labeled agile cones were arranged on the test path. Time was measured using an electronic timing system (TCI Timing System, Brower Timing Systems, Draper, UT, USA). After the examiner gave the start signal, the participant sprinted forward at 10 m and touched the tip of the agile cone with his right hand. Then, the subjects moved laterally 5 m to the left and touched the tip of the left marking cone with their left hand. Thereafter, the participants changed direction and moved 10 m to the right to touch the tip of the right marking cone with their right hand. Then, they traversed 5 m to the left to touch the middle marker cone. At the end, the participants ran backward 10 m and passed the finish timing gate to complete the test (Figure 2A). The recorded time (accuracy: 10 msec) was the best time of 2 attempts, and the participants had a 3-min recovery interval between 2 attempts [26].

#### 2.6.5. Planned Agility

The planned agility test, which was developed to mimic the distances and displacements encountered during a basketball game, was performed on a test court 20 m in length and 3 m in width [27]. The planned agility test consisted of 4 parts: a linear sprint (4.50 m), two backward side slips (7.50 m) with a change of direction, two forward spins (6 m) with a change of direction, and a final linear sprint (2 m). Two electronic timing gates (TCI Timing System) were placed at the beginning and the end lines to record the total time, respectively (Figure 2B). The best time of two attempts was recorded for analyses, and the participants had a 3-min recovery time between the two sprint attempts.

#### 2.6.6. 20 m Yo-Yo Shuttle Run Test

Based on the procedures of Leger and Mercier, participants performed a 20 m shuttle run test to evaluate the participant’s maximum aerobic capacity [28]. A pre-recorded tape with acoustic signals was used to guide participants with appropriate running speed during the test. Each participant ran at a speed set by signals between 2 marked lines separated by 20 m. The sound signal frequency started at 8.5 km/h and increased by 0.5 km/h each minute. The speed of the signal increased until the participants no longer followed the signal speed. The predicted maximum oxygen uptake was calculated using the following formula: (VO_2_max; mL/kg/min) = 31.025 + 3.238 × speed (km/h) − 3.248 × Age (years) + 0.1536 × speed (km/h) × Age (years).

### 2.7. Profile of Mood State (POMS) and Subjective Fatigue Levels

The Profiles Mood State (POMS) is used to measure the mood state of athletes under specific training/competition states. The POMS questionnaire consists of 37 questions in seven aspects [20]. The sub-aspects of POMS consist of positive moods (vigor and self-esteem) and negative moods (confusion, fatigue, anger, tension, and depression). The POMS was evaluated according to the manual. The subjective fatigue levels were evaluated using the Checklist Individual Strength (CIS), which has been recognized as a reliable and valid instrument used to assess subjective fatigue status [29].

### 2.8. Pittsburgh Sleep Quality Index (PSQI)

The Chinese version of the Pittsburgh Sleep Quality Index (PSQI) [30] was used for this study. The PSQI questionnaire includes nine questions divided into seven aspects: sleep quality, sleep latency, sleep time, habitual sleep efficiency, sleep disorders, sleep medication use, and daytime dysfunction. A general score ≤ 5 represents better sleep quality. Participants completed the questionnaire based on their sleep status over one month before the questionnaire was completed.

### 2.9. Saliva Sample Collection and Salivary DHEA-S/Cortisol Analyses

Immunoenzymatic analyses (ELISA) kits for dehydroepiandrosterone sulfate (DHEA-S) and cortisol were used to determine differences in systemic stress coping capacity between the competitive season (CS) and off-season (OS) recovery periods. Fasting saliva samples were collected at 0700–0800 AM in the morning using a Sarstedt Salivette cotton swab (Fisher Scientific, Inc., Pittsburgh, PA, USA). Participants first rinsed their mouth with pure water 3 times (50 mL of pure water/time) and then put a Sarstedt Salivette cotton swab in their mouth for 3 min to stimulate saliva secretion. Then, the saliva-adsorbed cotton swab was placed in a collection vial, and the collected saliva sample was centrifuged (1500× *g*, 15 min) to remove particulate matter. All participants involved in this study completed the salivary sample collection procedure, and the clear supernatant was transferred into a collection vial and stored at −80 °C until analyzed. For the saliva analyses, the biomarker measurements of two participants were undetectable, and we obtained salivary biomarker results from seven participants in total. Salivary samples were assessed for DHEA-S levels (intra CV% = 7.8%; #IB79308, Immuno-Biological Laboratories, Inc., Hamburg, Germany) following the manufacturer’s instructions. Cortisol was measured using an enzyme-linked immunosorbent assay (ELISA) from a commercially available kit (intra CV% = 8.48%; #500360, Cayman Chemical Co., Ann Arbor, MI, USA).

### 2.10. Statistical Analysis

All datasets were analyzed and plotted using SPSS 25.0 software (IBM-SPSS version 25.0 (IBM Corp., Armonk, NY, USA) and GraphPad Prism 5.0 (GraphPad Software, La Jolla, CA, USA). All data were expressed as mean ± standard error (Mean ± S.E.M.), and the percent change (Δ%) of the measured parameters between the competition period and the off-season period was calculated using the formula = [(off-season value − competition period value)/(competition period value)] × 100%. The normality of all data was determined by passing the Shapiro–Wilk normality test before performing the *t*-test statistical test, and the effect size (ES) was calculated using Cohen’s d. The differences in anthropometric parameters, basketball-specific physical performance, shuttle-running aerobic capacity, sleep quality, mood state, and salivary hormone concentrations (cortisol and DHEA-S) were compared using paired t-tests. All data were expressed as mean ± standard error of the mean (mean ± S.E.M.), and the alpha values of statistical differences for all comparisons are set to 0.05.

## 3. Results

### 3.1. Physical Activity and Anthropometric Measurements

**Figure 3:**
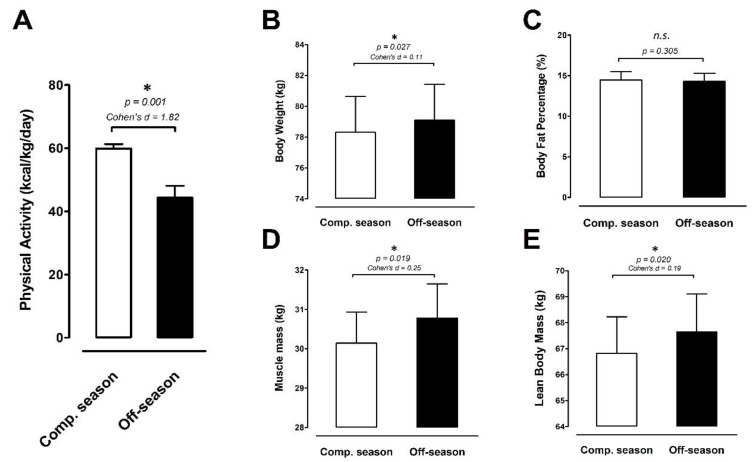
Physical activity and body composition. (**A**) Physical activity, (**B**) body weight, (**C**) body fat percentage, (**D**) muscle mass, and (**E**) lean body mass were measured at the competitive season and off-season (competitive season, open bar; off-season, black bar). Data are expressed as Mean ± S.E.M. * denotes significant difference between two phases (*p <* 0.05). Comp. season: competitive season (CS), off-season (OS).

A 3-day physical activity recall log (3d-PAL) assessed the participant’s physical activity level during the competitive season and off-season. The results are shown in Figure 3A. Physical activity levels during the off-season (44.3 ± 3.8 kcal/kg/day) were approximately ≈26% less than that during the competitive season (59.9 ± 1.4 kcal/kg/day) (ES 1.82; *p* = 0.001). Figure 3B–E shows anthropometric measurements, including body weight (BW, Figure 3B), percentage of body fat (%BF, Figure 3C), muscle mass (MM, Figure 3D), and lean body mass (LBM, Figure 3E) during the competitive season and off-season. Although the %BF did not show any difference between the competitive season and off-season periods, the BW (+1.0%; ES 0.11; *p* = 0.027), MM (+2.2%; ES 0.26; *p* = 0.019), and LBM (+1.2%; ES 0.19; *p* = 0.020) exhibited significant increases during the off-season compared with the competitive season in the elite adolescent basketball players.

### 3.2. Basketball-Specific Physical Performances

**Figure 4:**
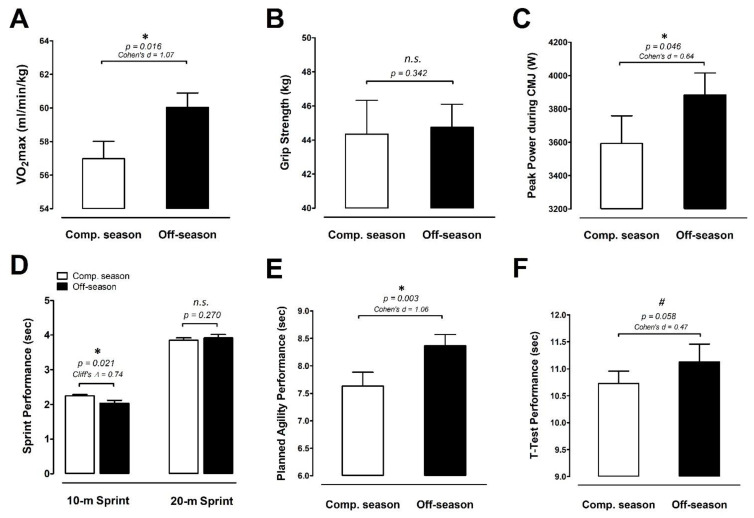
Basketball-specific physical performance. (**A**) VO_2_max, (**B**) grip strength, (**C**) Abalakov jump (AJ) peak power, (**D**) sprint performance, (**E**) basketball-specific planned and reactive performance, and (**F**) T-Test performance was measured at the competitive season and off-season (competitive season, open bar; off-season, black bar). Data are expressed as mean ± S.E.M. * denotes significant difference between two phases (*p <* 0.05). Comp. season: competitive season (CS), off-season (OS). # denotes approached significance (*p* < 0.075)

The estimated maximal oxygen uptake increased significantly by ≈5.4% during the off-season period compared with the competitive season (ES 1.07; *p* = 0.016; Figure 4A). However, there was no difference in dominant handgrip strength between the two periodic phases during the basketball training/competition cycle (Figure 4B). Figure 4C shows the peak Abalakov jump power (AJ). The peak AJ power is significantly greater in the off-season period than the competitive season (+8.1%; ES 0.64; *p* = 0.045). For the 10/20 m sprint, the results are shown in Figure 4D, and there were marked improvements in 10 m sprint time performance during the off-season compared to during the competitive season (sprint time: −9.8%; ES 1.10; *p* = 0.015). However, the overall 20 m sprint performance was not different between the off-season and the competitive season (*p* = 0.270). Figure 4E,F display the basketball-specific planned and reactive performance and T-Test performance, respectively. The basketball-specific planned and reactive performance declined significantly by increasing the completion time during the off-season compared with during the competitive season (+9.6%; ES 1.07; *p* = 0.003; Figure 4E). Although the T-Test performance displayed a similar pattern to that of the basketball-specific planned and reactive performance, there were only significant differences in T-Test performance between the off-season and competitive season periods (ES 0.47; *p* = 0.058; Figure 4F).

### 3.3. Responses of Subjective Fatigue and Salivary Hormones to Different Periodic Phases

**Figure 5:**
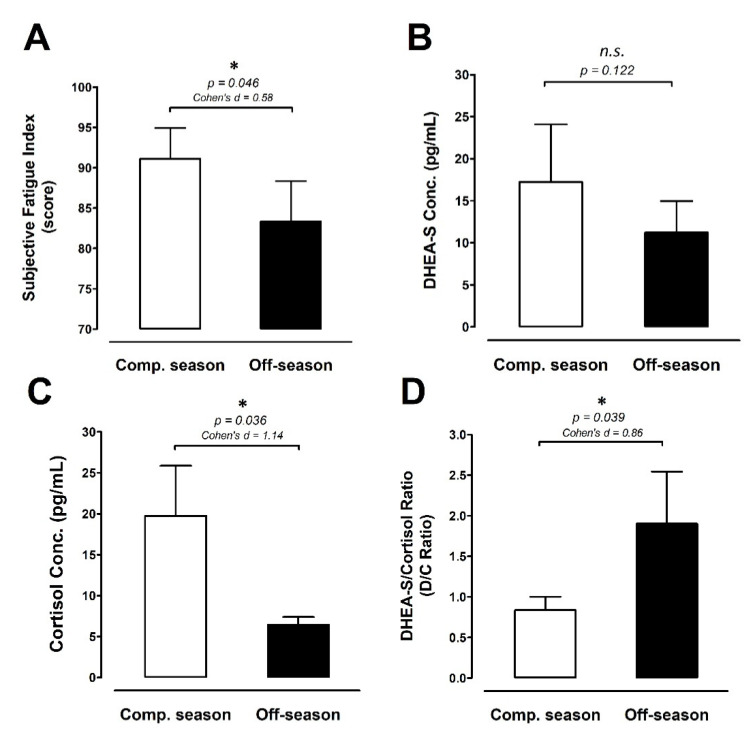
Fatigue and stress-related biomarkers. (**A**) Subjective fatigue index, (**B**) DHEA-S concentration, (**C**) cortisol, and (**D**) D/C ratio were measured at the competitive season and off-season (competitive season, open bar; off-season, black bar). Data are expressed as mean ± S.E.M. * denotes significant difference between two phases (*p <* 0.05). Comp. season: competitive season (CS), off-season (OS), D/C ratio: DHEA-S/Cortisol ratio.

Figure 5A displays subjective fatigue levels. The subjective fatigue level was significantly lower during the off-season period than during the competitive season (−8.5%; ES 0.58; *p* = 0.046). The salivary DHEA-S levels are shown in Figure 5B. There was no difference in DHEA-S between the competitive season and off-season periods. Conversely, the salivary cortisol level was significantly lower during the off-season period compared with the competitive season (–67.4%; ES 1.14; *p* = 0.036; Figure 5C). Furthermore, the DHEA-S-to-cortisol ratio (D/C ratio) is illustrated in Figure 5D. During the off-season, the D/C ratio rose significantly by approximately ≈127.5% above the competitive preparation season level (D/C ratio: ES 0.86; *p* = 0.039).

### 3.4. Changes in Sleep Quality and Mood States between Different Periodic Phases

**Figure 6:**
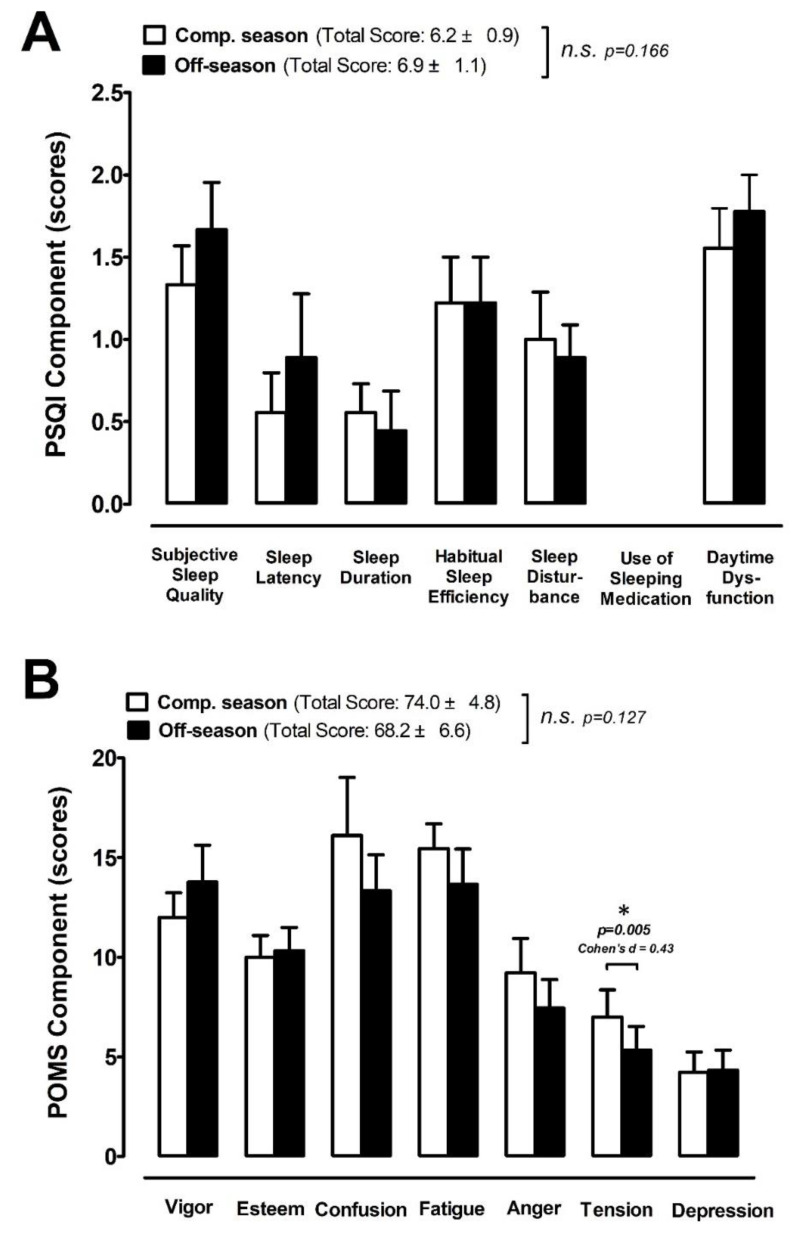
Sleep quality and mood state. (**A**) Pittsburgh Sleep Quality Index (PSQI) and (**B**) Profile of Mood State (POMS) were measured at the competitive season and off-season (competitive season, open bar; off-season, black bar). Data are expressed as Mean ± S.E.M. * denotes significant difference between two phases (*p <* 0.05). Comp. season: competitive season (CS), off-season (OS).

The sleep quality assessed using the PSQI questionnaire is shown in Figure 6A. No difference in the overall sleep quality score was observed between the competitive and off-season phases (CS: 6.2 ± 0.9 vs. off-season: 6.9 ± 1.1 AU; *p* = 0.166) in the male elite basketball players. There were no differences between phases for subjective sleep quality, sleep latency, sleep duration, habitual sleep efficiency, sleep disturbance, use of sleeping medication, and daytime dysfunction (CS vs. OS; *p* > 0.05). The POMS score results between two phases are shown in Figure 6B. There was no difference in overall mood states assessed using POMS between the competitive and OS phases (CS: 74.0 ± 4.8 vs. OS: 68.2 ± 6.6 AU; *p* = 0.127) in this population. However, the competitive season presented a higher score for tension (CS: 7.0 ± 1.4 vs. OS: 5.3 ± 1.2 AU; +24.3%; ES 0.43; *p* = 0.005) compared with OS phase. There were no differences between phases for vigor, esteem, confusion, fatigue, anger, and depression (CS vs. OS; *p* > 0.05).

## 4. Discussion

We originally hypothesized that sports performance, conditioning, and body composition would be better during the competitive season, and that the mood state and sleep quality would be superior during the off-season period. Interestingly, parts of our findings are contrary to our hypotheses. It is important to note that our study design is observational in nature, with no control group. Our observational study focuses primarily on making inferences about the effects of an “exposure” on participants [31] (i.e., basketball training/competition/off-season periods), where the periodic stages in this study were observed and classified by the investigators but not through manipulation. Moreover, our observational study involves direct observation of individuals in their natural environment, and this inevitable confounding could be a major challenge for observational studies. The primary findings of this study were as follows: (1) Body composition parameters (i.e., muscle mass and lean body mass) exhibited a greater positive training adaption during the off-season. (2) The fundamental physical capacity (i.e., aerobic capacity, peak AJ power, 10 m sprint performance), but not basketball-specific agility, was greater during the off-season compared to the competitive season. (3) Based on DHEA-S/cortisol ratios, the off-season presented relatively lower physiological stress. (4) There were no differences in overall sleep quality and mood states between the competitive season and off-season periods, but the tension sub-element was lower during the off-season. The significance of this present investigation was that contrary to our hypotheses, the basketball-specific sport performance and body composition parameters were relatively greater during the off-season than during competitive season in these adolescent basketball players; furthermore, these elite student–athletes seemed to be perturbed by poor sleep quality (PSQI > 5.0) across different training/competition seasons.

Physical activity levels during the competitive season were approximately ≈26% higher than that during the off-season, while the BW, MM, and LBM all exhibited significant increases during the off-season compared with the competitive season in these elite adolescent basketball players. The peak AJ power and 10 m sprint time performance were significantly greater in the off-season period than the competitive season (Figure 4), suggesting that the enhancement of jump and speed is partly due to changes in body composition. However, agility ability tended to decline during the off-season compared with the competitive season. This finding was intriguing; thus, we speculate that the poor agility performance was due to the lack of basketball-specific agility training during the off-season period. This is likely because the training regimen primarily focused on maintaining/recovering fundamental physical capacity rather than the specific sport skill training during the off-season [32,33]. Taken together, the dramatic decrease in training load (Figure 3A) during the off-season may have contributed to the positive adaptions on body composition and fundamental sport-specific performance in these elite male adolescent basketball players.

We observed a decreasing but non-significant transition trend in salivary DHEA-S concentrations between the competitive season and off-season periods (Figure 5B). The DHEA-S responses to different training periods in our study were inconsistent with several previous studies [34,35]. However, our findings are supported by those reported in several other studies [22,36,37]. Nevertheless, we found that salivary cortisol levels were significantly lower during the off-season period compared with the competitive season, and the D/C ratio was higher in the off-season than in the competitive season (Figure 5C,D). These results suggest that the substantial physiological stress caused by extreme competition/training load during the competitive season could have led to a greater decline in D/C ratio compared with the off-season, indicating that the greater stress/catabolic responses occurred during the competitive season [20,21]. Hypothalamus–pituitary–adrenal (HPA) axis activation during high-intensity exercise/competition represents physiological stress responses to the strain of muscular exercise, thereby increasing the release of cortisol and reflecting the metabolic stress [38,39]. Additionally, we also observed a much less subjective fatigue index during the off-season period than during the competitive season (Figure 5A). These results imply that the physiological and subjective fatigue stress responses gradually declined from competitive season to off-season periods, which is in line with our present finding that the POMS tension element was significantly lower in the off-season (Figure 6B). In addition, the mental and physical stress conversion process was also demonstrated by psychological measures in elite athletes [40,41].

On the other hand, the dose–response relationship between increased training load and DHEA-S/C has been identified [42], and the relationship between negative mood and D/C ratio has been proposed in young male populations [43]. Previous evidence reveals that a chronic imbalance between stress (including training, competition, and non-training stress factors) and recovery (e.g., sleep) can lead to negative changes in physiological growth and mood states in adolescents [44,45]. Although the overall POMS mood score was not different between the two investigated seasons, a remarkably higher score for the tension sub-element was observed during the competitive season (Figure 6B). These results indicated that adolescent basketball players still experienced greater mental stress during the competitive season, and such mental responses were also reflected by a greater salivary cortisol response (Figure 5C).

Sleep quality and habitual sleep have been reported to decrease in adolescent populations [46]. In this regard, coaches should make adolescent student–athletes aware of the effects of insufficient sleep on their overall development [47]. The literature shows that chronic sleep deprivation can impair long-term athletic and academic achievement and may result in increased risks for serious pathophysiologies, illness, sport injuries, and other health-related issues among this population [13,48]. In this study, we found that the PSQI scores of two different phases presented with bad sleep quality (the score of PSQI > 5), but there was no difference between the competitive season and off-season in the sub-elements of PSQI, including sleep quality, sleep latency, sleep duration, habitual sleep efficiency, sleep disturbance, and daytime dysfunction (Figure 6A). Although bad mood states were associated with poor sleep quality in elite athletes [49], such a pattern was not observed in this present investigation. These athletes experienced poor sleep quality (PSQI > 5) across both training/competition periods, which might create a ceiling effect on the perturbations in mood states.

However, it has to be noted that sleep deprivation is a common phenomenon among athletes during intense training and/or prior to competition, and sleep disturbance has been reported to magnify mental challenges, which were evaluated by the perceived difficulty of the options offered and the difficulty of the task selected, in competitive adolescent athletes [18]. Likewise, Poussel et al. (2014) investigate the sleep patterns and academic performance among adolescent elite athletes (mean age: 15.7 years) and found that disturbed sleep patterns affect academic performance in young elite athletes [50]. From this aspect, teachers, athletic trainers, physicians, and/or other professionals dealing with adolescent elite athletes should pay particular attention to this condition; poor sleep patterns induced by intensive training may negatively impact academic and athletic performance. Therefore, further research and consideration are warranted to establish knowledge about the interactions between sleep and academic performance.

Lastly, since there is substantial mental and physical growth during adolescents, it is critical to closely monitor stress-induced responses brought on by intense sports training/competitions to maintain normal physiological and mental development in youth athletes. Therefore, our findings demonstrated that changing patterns of sleep quality, mood state, and stress-related biomarkers between the competitive and off-season periods provide fundamental evidence for coaches and team sport scientists to better modify appropriate training/recovery programs for adolescent athletes.

### 4.1. Practical Applications

The current study reports that physiological stress is significantly higher during the season than in the off-season. Sleep quality is poor during this period. Given these findings, adolescent athletes face significant stresses that may impact their physical growth/training development. Therefore, the practical implications of this study suggest that it is necessary to closely monitor stress levels resulted from intense athletic training/competition by using these fatigue and mental questionnaires to help maintain their normal physiological and psychological development, and the coaches could be better to react and modify athletic training or recovery plans for these adolescent athletes. Coaches and trainers working with adolescent athletes may use this information to best prescribe the appropriate training/recovery strategies for their athletes.

### 4.2. Study Limitations

One of the major limitations in this study was that the participants were limited to high school Division I basketball players. Thus, there was a narrow range of age (i.e., 15 to 18 years old), and all the athletes trained and competed on the same high school varsity team. In this regard, we did our best to eliminate as many potential confounding factors as possible in our study design, including the fact that athletes were accommodated in student dorms and that coaches standardized dietary guidance and developed a monitored basketball-specific training program. Despite our efforts to control for possible interfering factors, we still cannot completely rule out the possibility of Type II errors due to the relatively small sample size. Another limitation of this study was the lack of a control group. However, the primary population of this study was national-level elite high school boys’ basketball players (top eight in the country), which is an inherently unique group, and participant number could be limited. Therefore, it may not be appropriate to generalize our findings to other sports or non-athletic adolescent students. For the body composition measurement, we did carefully control all the possible confounding factors that might affect the BIA assessment, but there are still several technical limitations when using BIA compared with using other gold-standard measurements (e.g., dual-energy X-ray absorptiometry or underwater weighing). Moreover, although we documented the training program schedule of all athletes and all training was performed and monitored by the coaches, there was one limitation in that we did not record the rate of perceived exertion of each training session in this study; therefore, it is suggested that future long-term monitoring studies may be required to record the subjective efforts or training impulse (TRIMP) of each training session, which may help provide more objective data to support the interpretation of the obtained results. Lastly, although it is unequivocal that academic requirements are a major source of stress for adolescent students and student–athletes alike, stress brought by school work was not assessed in the present study. The stress interaction between academics and sports warrants further investigation.

## 5. Conclusions

In conclusion, this study demonstrates that muscle mass, lean body mass, aerobic capacity, peak AJ power, and 10 m sprint performance of elite adolescent high-school basketball players were greater during the off-season than during the competitive season. We also found that the D/C ratio and tension were relatively lower during the off-season period. These results suggest that during the off-season period, greater training adaptations in fundamental physical sport performance and muscle mass might be the result of the positive anabolic/catabolic balance and lower mental stress in these elite adolescent athletes. We suggest that the coaches and sport science professionals should closely monitor athletes’ training states across varied training/competition periods by using these simply used questionnaires to better react to modifying training or recovery plans. Our current findings provide fundamental evidence for coaches and team sports scientists to help modify appropriate training/recovery programs for youth student–athletes.

## Figures and Tables

**Table 1:** Anthropometric data of participants.

Variables	N = 9
Age (year)	16.1 ± 0.2
Height (cm)	185.9 ± 1.5
Weight (kg)	78.3 ± 1.4
Body fat percentage (%)	14.5 ± 1.0
Muscle mass (kg)	30.1 ± 0.8

**Table 2:** Training programs for the competitive season and off-season.

	Competitive Season (CS)	Off-Season (OS)
**Total Training Hours per week**	**20 h**	**14 h**
**Basic physical training** Endurance run, shuttle run, personal practice (dribbling, passing, shutting, cut, crossover, layup, screen)	**6 h/week** ✓ 60 min/session/day ✓ 6 sessions/week	**6 h/week** ✓ 60 min/session/day ✓ 6 sessions/week
**Basketball-specific training** Motion offense, defense, one on one, two on two, three on three, team practice, etc.	**6 h/week** ✓ 60 min/session/day ✓ 6 sessions/week	**3 h/week** ✓ 60 min/session/day ✓ 3 sessions/week
**Strength and Conditioning** Weight/training, agility training, speed training, plyometric training, etc.	**3 h/week** ✓ 60 min/session/day ✓ 3 sessions/week	**3 h/week** ✓ 60 min/session/day ✓ 3 sessions/week
**Practice Tournament** (Competitions or practice games)	**5 h/week** (Competition and practice)	**2 h/week** (Practice games only)
**Coach rating training intensity** ✓ Physical Challenge (PC) ✓ Skill-Specific Challenge (SC)	✓ PC: 6 scores ✓ SC: 8 scores	✓ PC: 7 scores ✓ SC: 3 scores

Note: The Coach rating training intensity (1–10 scale) was rated by the head coach according to the challenge levels of training (scoring level: 1-to-10 scale; e.g., 1: extremely easy; 10: extremely hard).

## Data Availability

The data presented in this study are available upon request from the corresponding author.
